# Supplementary material for: mosGILT controls innate immunity and germ cell development in Anopheles gambiae
Source: BMC Genomics. 2024 Jan 9;25:42. doi: 10.1186/s12864-023-09887-0 (PMC10775533; doi:10.1186/s12864-023-09887-0)
Supplement: Supplementary file 1 — Additional file 1: Supplementary Figure 1. Distribution of Raw counts in all Anopheles samples. Supplementary Figure 2. Key statistics of Ovary samples. Supplementary Figure 3. Key statistics of whole-body samples. Supplementary Figure 4. Key statistics of Fatbody samples. Supplementary Figure 5. Pathway enrichment analysis using the g:Profiler online tool. Table provides comprehensive information related to the selected term, including the data source, term ID, term name, and corresponding p-value. Abbreviations: GO:BP (Gene Ontology: Biological Process); GO:CC (GO: Cellular Component); GO:MF (GO: Molecular Function). Supplementary Figure 6. Protein-Protein Interaction (PPI) network: The interaction Network of DEGs in the Ovary of mosGILTnull mosquitoes was created using the STRING database and imported into Cytoscape. The k clustering method was used to identify clusters and key nodes in this network. [file 12864_2023_9887_MOESM1_ESM.pdf]

Supplementary Figure 1

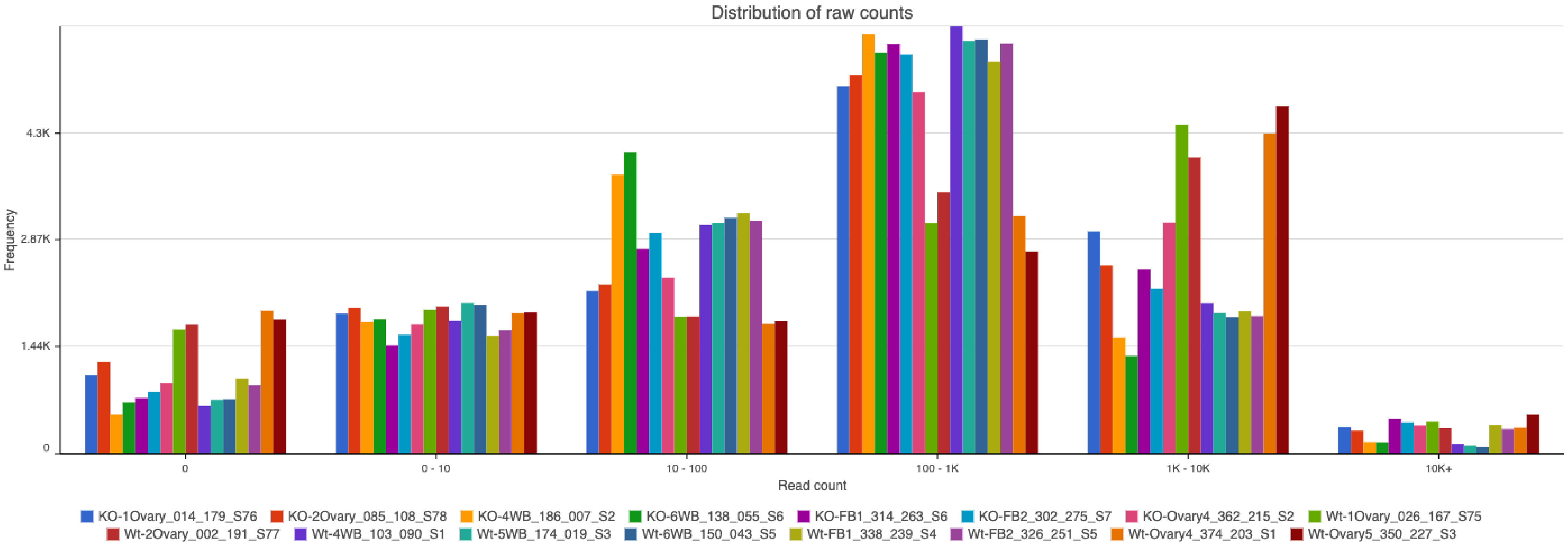

Supplementary Figure 1: Distribution of Raw counts in all Anopheles samples

Supplementary Figure 2

Summary of reads quantified to aga - gambiae

Optional columns

Sample name	Total reads	Fully within an exon	Partly within an exon	Fully within an intron	Fully intergenic	Incompatible paired-end	Compatible junctions	Total junctions	View
KO-1Ovary_014_179_S76	26,318,938.00	78.73%	3.13%	3.99%	8.70%	5.45%	3,400,774.00	4,370,374.00	
KO-2Ovary_085_108_S78	22,776,096.00	78.46%	3.11%	3.82%	9.06%	5.55%	2,967,114.00	3,805,961.00	
KO-Ovary4_362_215_S2	27,398,899.00	78.03%	4.24%	3.98%	6.96%	6.80%	5,323,700.00	6,925,909.00	
Wt-1Ovary_026_167_S75	28,468,342.00	82.18%	3.05%	2.39%	6.89%	5.49%	3,601,721.00	4,561,748.00	
Wt-2Ovary_002_191_S77	23,381,670.00	81.93%	3.14%	2.38%	7.00%	5.55%	2,876,711.00	3,661,624.00	
Wt-Ovary4_374_203_S1	25,073,627.00	81.30%	4.52%	2.09%	5.93%	6.16%	4,222,895.00	5,357,611.00	
Wt-Ovary5_350_227_S3	32,241,676.00	81.89%	4.42%	1.83%	5.58%	6.28%	5,805,263.00	7,427,826.00	
Average	26,522,749.71	80.42%	3.69%	2.89%	7.08%	5.92%	4,028,311.14	5,158,721.86	

Rows per page 25 (1 of 1) Download

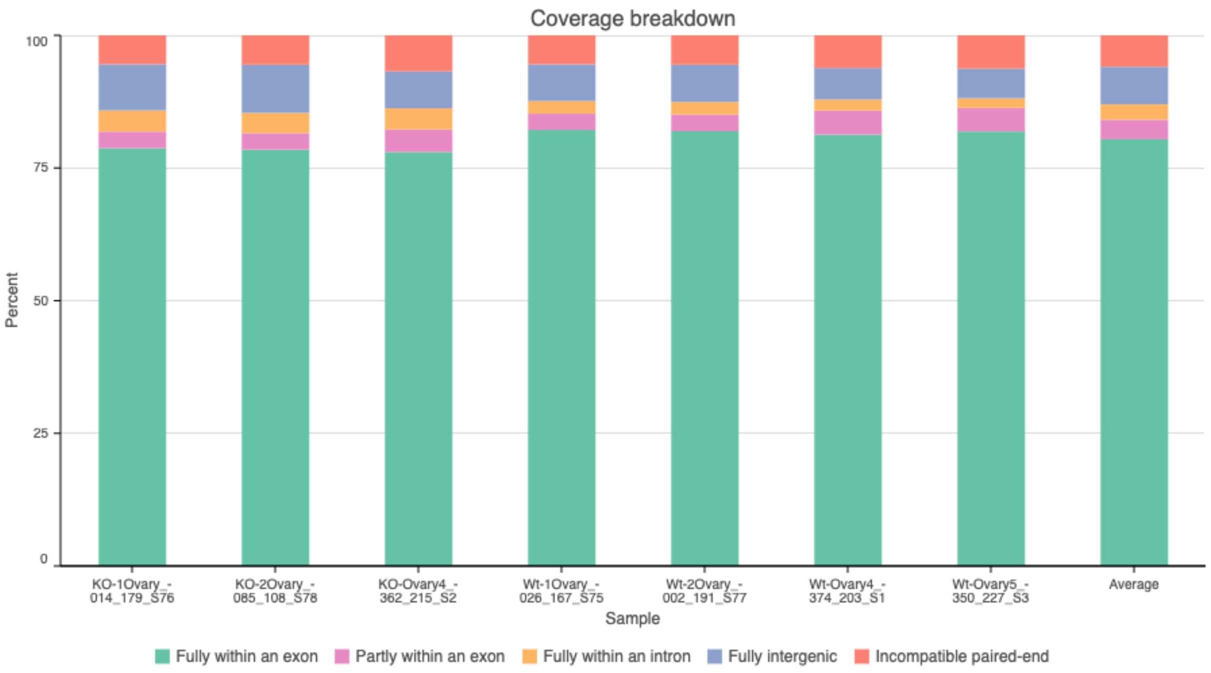

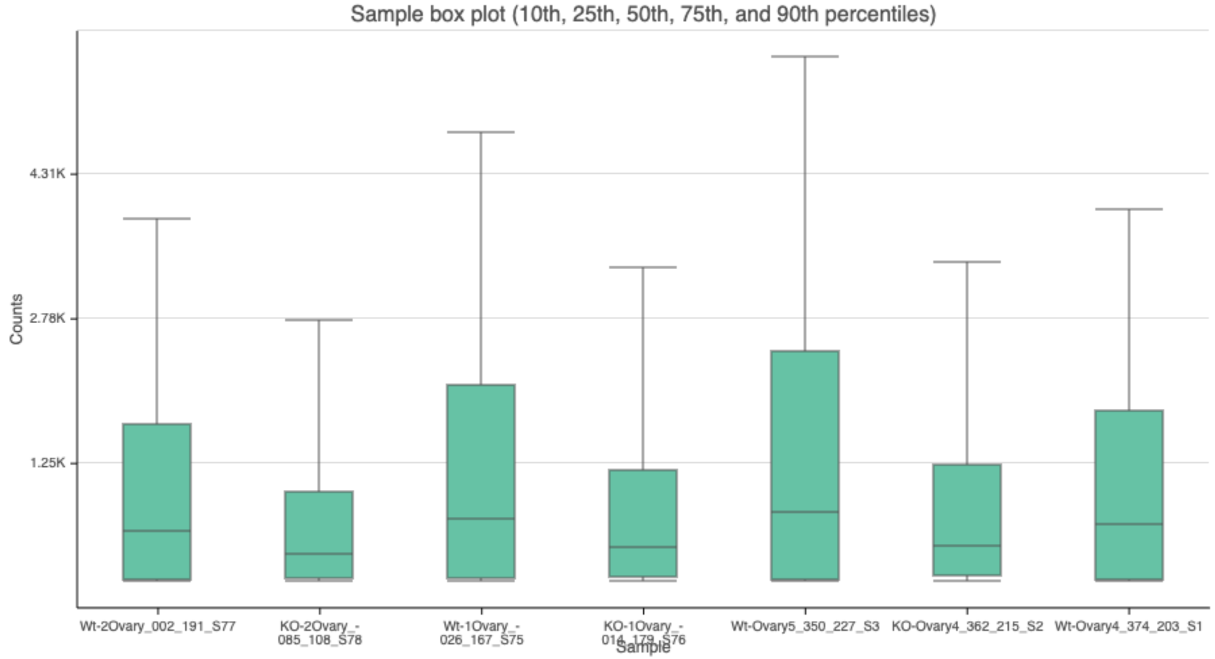

Supplementary Figure 2: Key statistics of Ovary samples

Supplementary Figure 3

Optional columns

Sample name	Total reads	Fully within a feature	Partly within a feature	Not in a feature	Incompatible paired-end	Compatible junctions	Total junctions	View
KO-4WB_186_007_S2	10,712,966.00	99.74%	0.10%	0.00%	0.16%	2,252,491.00	2,280,500.00	
KO-6WB_138_055_S6	10,799,903.00	99.76%	0.10%	0.00%	0.14%	1,996,089.00	2,021,807.00	
Wt-4WB_103_090_S1	12,371,793.00	99.77%	0.09%	0.00%	0.14%	2,279,888.00	2,308,628.00	
Wt-5WB_174_019_S3	9,958,040.00	99.72%	0.11%	0.00%	0.17%	2,102,655.00	2,130,788.00	
Wt-6WB_150_043_S5	9,436,270.00	99.72%	0.11%	0.00%	0.17%	1,801,246.00	1,827,209.00	
Average	10,655,794.40	99.74%	0.10%	0.00%	0.15%	2,086,473.80	2,113,786.40	

Rows per page 25 (1 of 1) Download

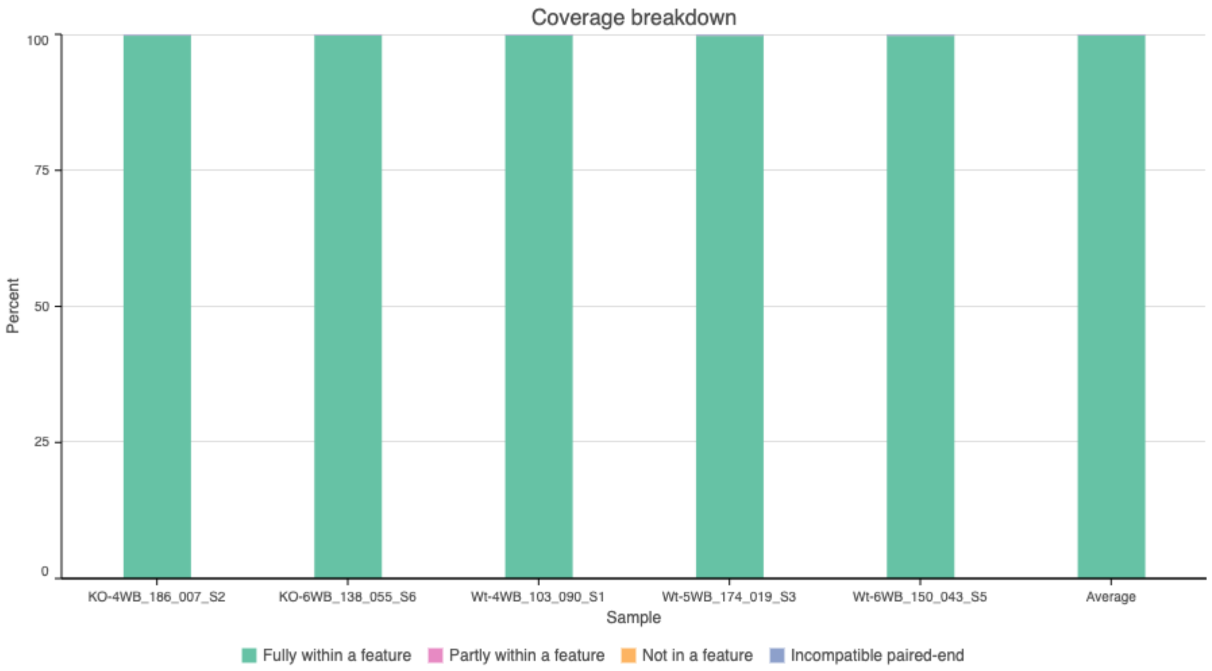

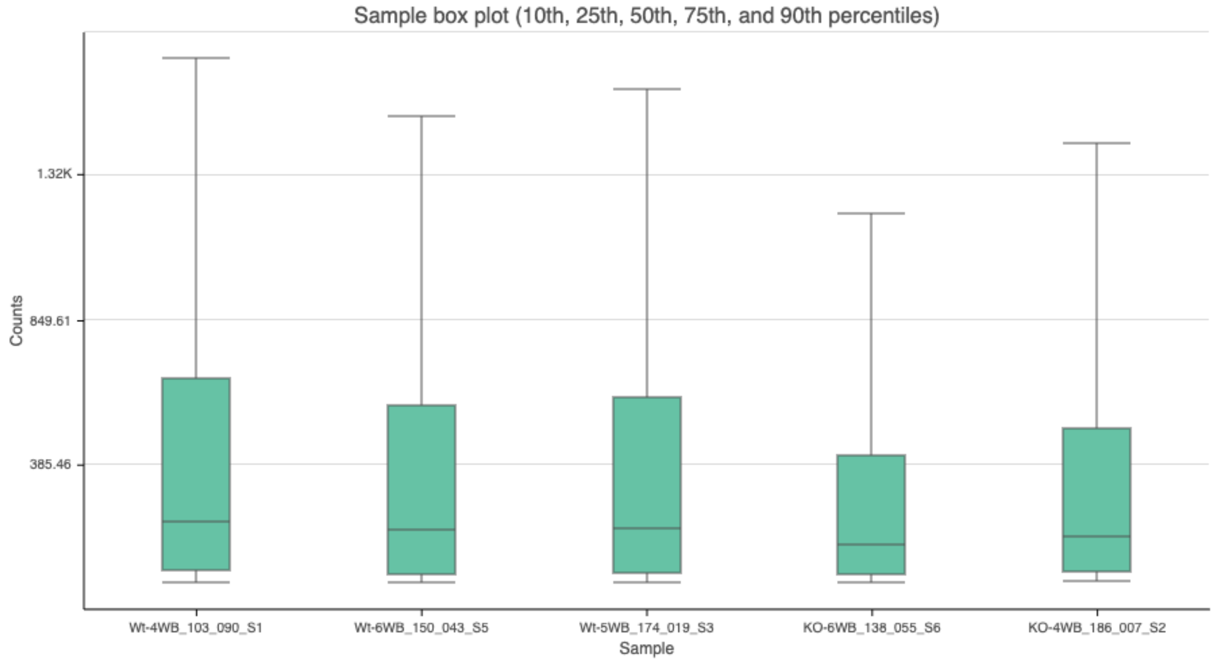

Supplementary Figure 3: Key statistics of whole-body samples

Supplementary Figure 4

Optional columns

Sample name	Total reads	Fully within a feature	Partly within a feature	Not in a feature	Incompatible paired-end	Compatible junctions	Total junctions	View
KO-FB1_314_263_S6	29,746,900.00	82.95%	3.68%	7.27%	6.10%	5,293,676.00	6,685,353.00	
KO-FB2_302_275_S7	30,136,606.00	84.56%	3.54%	5.38%	6.53%	5,591,036.00	7,102,196.00	
Wt-FB1_338_239_S4	28,560,821.00	83.39%	3.66%	6.50%	6.44%	4,362,502.00	5,486,092.00	
Wt-FB2_326_251_S5	26,714,071.00	83.84%	3.68%	5.79%	6.69%	4,050,396.00	5,172,691.00	
Average	28,789,599.50	83.69%	3.64%	6.24%	6.43%	4,824,402.50	6,111,583.00	

Rows per page 25 (1 of 1) Download

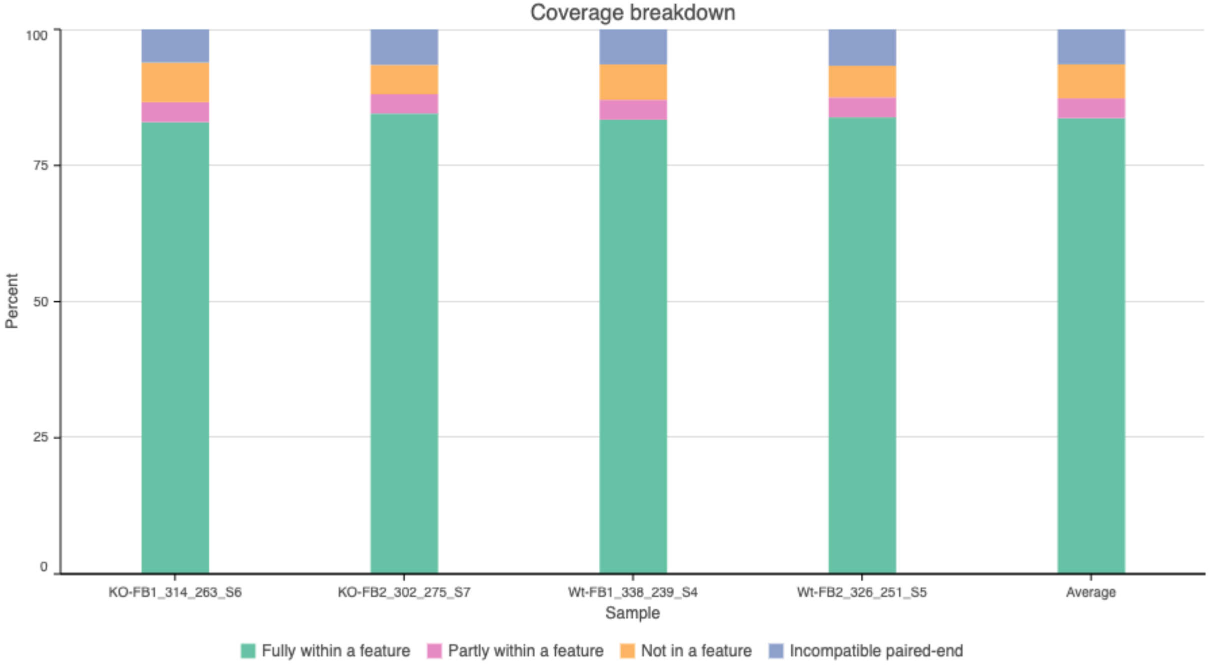

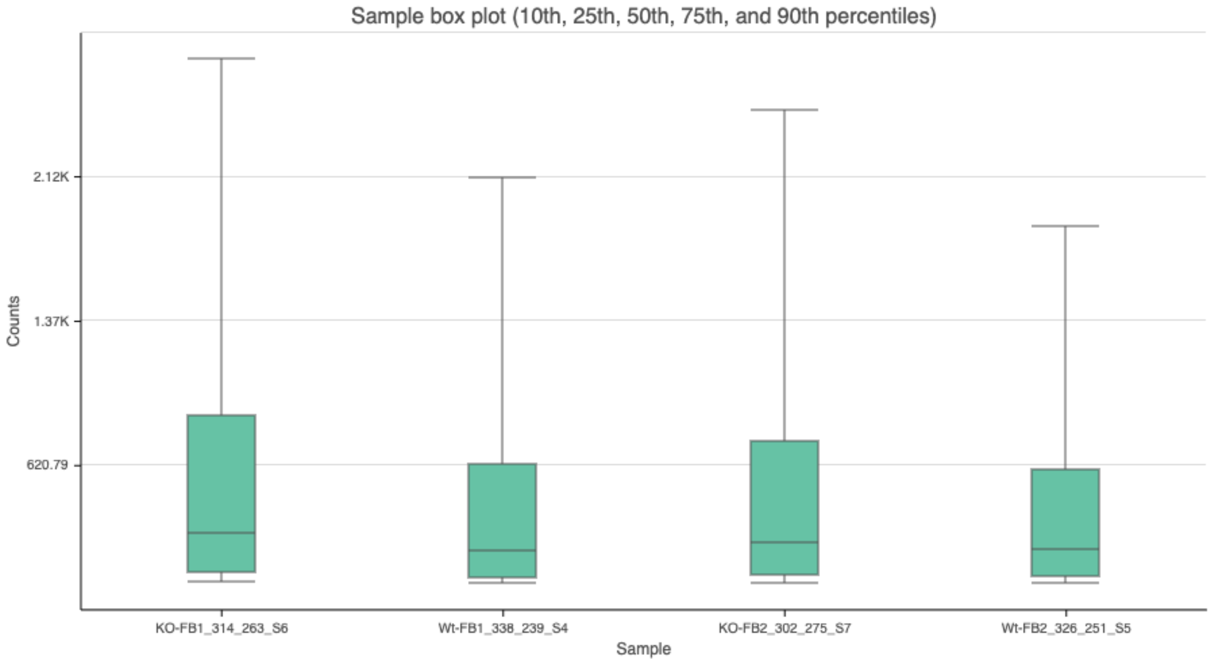

Supplementary Figure 4: Key statistics of Fatbody samples

Supplementary Figure 5

ID	Source	Term ID	Term Name	p_{adj} (query_1)
1	GO:MF	GO:0003676	nucleic acid binding	3.175×10^{-101}
2	GO:MF	GO:0005515	protein binding	5.581×10^{-24}
3	GO:MF	GO:0015453	oxidoreduction-driven active transmembrane t...	4.107×10^{-9}
4	GO:MF	GO:0015078	proton transmembrane transporter activity	3.330×10^{-8}
5	GO:MF	GO:0003688	DNA replication origin binding	4.061×10^{-5}
6	GO:MF	GO:0003712	transcription coregulator activity	6.201×10^{-4}
7	GO:MF	GO:0140993	histone modifying activity	1.081×10^{-3}
8	GO:MF	GO:0017056	structural constituent of nuclear pore	2.425×10^{-3}
9	GO:MF	GO:0019887	protein kinase regulator activity	4.602×10^{-3}
10	GO:MF	GO:0046933	proton-transporting ATP synthase activity, rota...	3.527×10^{-2}
11	GO:BP	GO:0006139	nucleobase-containing compound metabolic p...	1.661×10^{-94}
12	GO:BP	GO:0007049	cell cycle	1.334×10^{-15}
13	GO:BP	GO:0009060	aerobic respiration	9.462×10^{-14}
14	GO:BP	GO:0006913	nucleocytoplasmic transport	5.226×10^{-12}
15	GO:BP	GO:0046034	ATP metabolic process	3.482×10^{-4}
16	GO:BP	GO:0098660	inorganic ion transmembrane transport	1.117×10^{-3}
17	GO:BP	GO:0070647	protein modification by small protein conjugati...	2.003×10^{-3}
18	GO:BP	GO:0071824	protein-DNA complex subunit organization	4.031×10^{-3}
19	GO:BP	GO:0098655	monoatomic cation transmembrane transport	4.079×10^{-3}
20	GO:BP	GO:1902652	secondary alcohol metabolic process	6.149×10^{-3}
21	GO:CC	GO:0005634	nucleus	2.120×10^{-126}
22	GO:CC	GO:0070469	respirasome	9.751×10^{-16}
23	GO:CC	GO:0016469	proton-transporting two-sector ATPase complex	3.651×10^{-5}
24	GO:CC	GO:0005925	focal adhesion	3.592×10^{-2}

Supplementary Figure 5: Pathway enrichment analysis using the g:Profiler online tool. Table provides comprehensive information related to the selected term, including the data source, term ID, term name, and corresponding p-value. Abbreviations: GO:BP (Gene Ontology: Biological Process); GO:CC (GO: Cellular Component); GO:MF (GO: Molecular Function).

Supplementary Figure 6

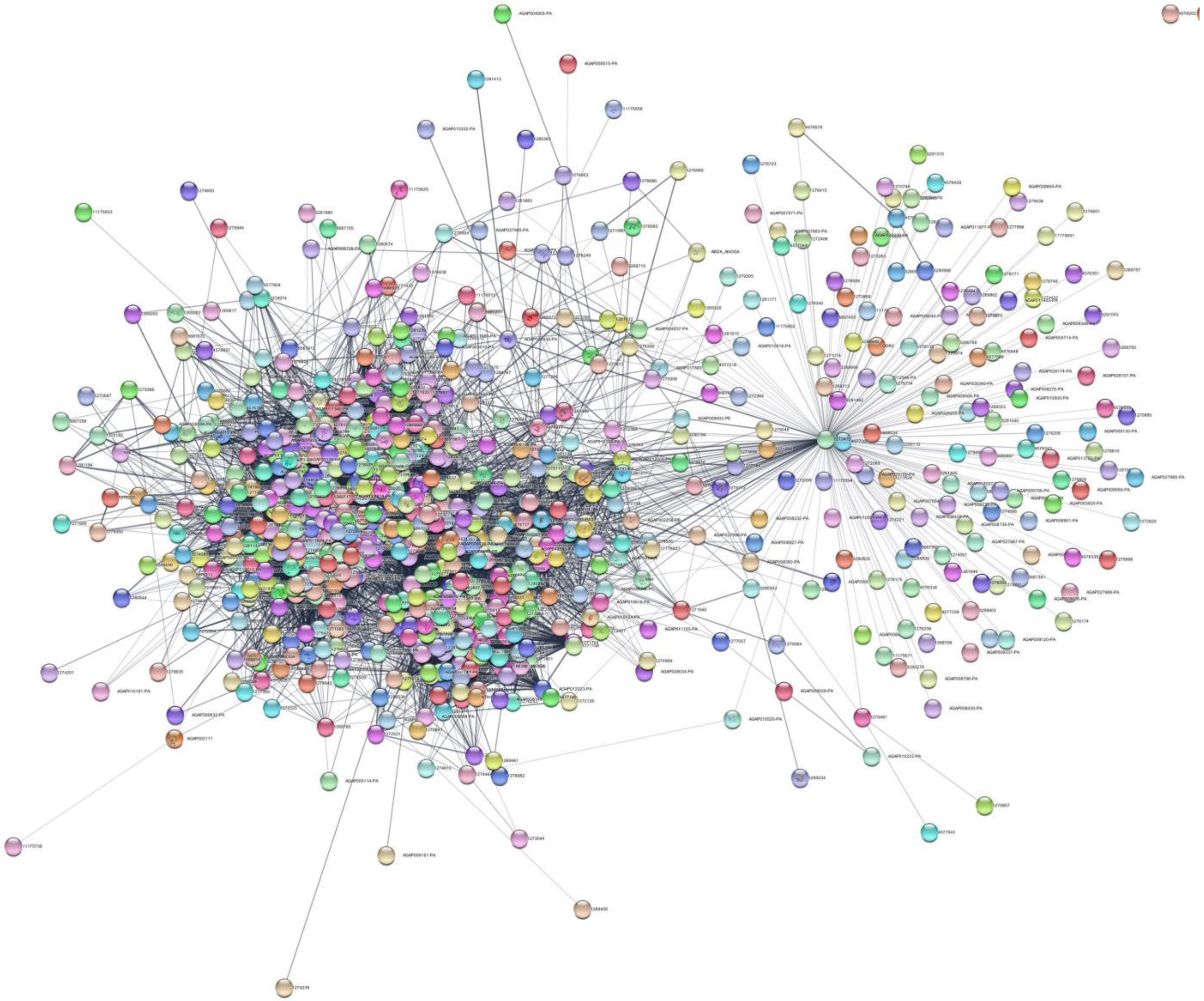

Supplementary Figure 6: Protein-Protein Interaction (PPI) network: The interaction Network of DEGs in the Ovary of *mosGILT*^{null} mosquitoes was created using the STRING database and imported into Cytoscape. The k clustering method was used to identify clusters and key nodes in this network.
